# Long term rebaudioside A treatment does not alter circadian activity rhythms, adiposity, or insulin action in male mice

**DOI:** 10.1371/journal.pone.0177138

**Published:** 2017-05-05

**Authors:** Thomas H. Reynolds, Rachelle A. Soriano, Obadi A. Obadi, Stanley Murkland, Bernard Possidente

**Affiliations:** Department of Health and Exercise Sciences and Department of Biology, Skidmore College, Saratoga Springs, NY, United States of America; Medical University of Vienna, AUSTRIA

## Abstract

Obesity is a major public health problem that is highly associated with insulin resistance and type 2 diabetes, two conditions associated with circadian disruption. To date, dieting is one of the only interventions that result in substantial weight loss, but restricting caloric intake is difficult to maintain long-term. The use of artificial sweeteners, particularly in individuals that consume sugar sweetened beverages (energy drinks, soda), can reduce caloric intake and possibly facilitate weight loss. The purpose of the present study was to examine the effects of the artificial sweetener, rebaudioside A (Reb-A), on circadian rhythms, in vivo insulin action, and the susceptibility to diet-induced obesity. Six month old male C57BL/6 mice were assigned to a control or Reb-A (0.1% Reb-A supplemented drinking water) group for six months. Circadian wheel running rhythms, body weight, caloric intake, insulin action, and susceptibility to diet-induced obesity were assessed. Time of peak physical activity under a 12:12 light-dark (LD) cycle, mean activity levels, and circadian period in constant dark were not significantly different in mice that consumed Reb-A supplemented water compared to normal drinking water, indicating that circadian rhythms and biological clock function were unaltered. Although wheel running significantly reduced body weight in both Reb-A and control mice (P = 0.0001), consuming Reb-A supplemented water did not alter the changes in body weight following wheel running (P = 0.916). *In vivo* insulin action, as assessed by glucose, insulin, and pyruvate tolerance tests, was not different between mice that consumed Reb-A treated water compared to normal drinking water. Finally, Reb-A does not appear to change the susceptibility to diet-induced obesity as both groups of mice gained similar amounts of body weight when placed on a high fat diet. Our results indicate that consuming Reb-A supplemented water does not promote circadian disruption, insulin resistance, or obesity.

## Introduction

Obesity is an increasingly prevalent metabolic disease [1] that is associated with elevated risk of developing type 2 diabetes, a disease that affects approximately 24 million Americans [2] and adds an estimated $245 billion to total health care costs in the United States [3]. Despite the astounding prevalence of obesity, there are few effective interventions that promote weight loss. Regular exercise improves overall health, but the caloric deficit produced by a vigorous bout of exercise is not substantial enough to result in weight loss. Although restricting energy intake can result in significant weight loss, dieting is difficult to maintain as individuals typically regain their lost weight within 1–3 years [4–7].

The consumption of sugar sweetened beverages likely contributes to the obesity epidemic [8]. Artificial sweeteners such as saccharin, aspartame, stevia, and sucralose are thought to provide a method to reduce energy intake without reducing food intake, thereby creating an energy deficit that would result in weight loss and avoiding the homeostatic response that triggers weight regain. However, recently it has been suggested that consuming artificially sweetened beverages might actually promote obesity [9] and the consumption of saccharin may cause insulin resistance [10]. One mechanism by which artificial sweeteners may lead to weight gain and impaired insulin action is by altering circadian rhythms. This idea is supported by the observation that obesity and insulin resistance are associated with circadian disruption [11]. Recently, saccharin was shown to disrupt circadian sleep-wake cycles and cause physical inactivity in mice [12]. Since obesity and insulin resistance are associated with circadian disruption [11], it is feasible that artificial sweeteners may promote weight gain by altering circadian rhythms.

The physiological effects of consuming the artificial sweetener rebaudioside A (Reb-A), a metabolite of steviol glycosides synthesis in the leaves of Stevia rebaudiana Bertoni [13], has not been studied extensively. To date, no studies have examined whether or not Reb-A consumption results in circadian disruption, impairs glucose metabolism, or promotes weight gain. Therefore, the purpose of the present study is to determine if consuming Reb-A disrupts circadian rhythms, causes insulin resistance, and increases the susceptibility of diet-induced obesity.

## Material and methods

### Animals

This study was approved by Skidmore College’s Institutional Animal Care and Used Committee (IACUC). Twenty C57BL6/J male mice (~1 month old) were purchased from the Jackson Laboratory (Bar Harbor, ME) and housed in our animal facility for five months in standard mouse cages. At six months of age, mice were randomly assigned to either a rebaudioside A (Reb-A) group (0.1% Reb-A added to drinking water) or control group (regular drinking water) and placed in wheel running cages for 32 days. Following an unmonitored one-week acclimatization period (days 1–7), wheel running activity was monitored in the 12 hour light-dark (12:12 LD) cycle for 14 days (days 8–22). During the final 10 days of wheel running (days 23–32) mice were in constant darkness (DD). Following the assessment of wheel running activity, mice were returned to standard mouse cages and allowed to recover for three months before measuring *in vivo* insulin action. During this three month recovery period mice continued to consume either 0.1% Reb-A water or normal drinking water. Mice were fed a standard rodent diet ad libitum (Purina RMH 3000) unless noted otherwise. Mice were euthanized by an overdose of sodium pentobarbital delivered by intraperitoneal injection.

### Circadian rhythms for wheel running activity

Following a one-week acclimatization period of unmonitored wheel running (days 1–7), circadian rhythms for wheel-running activity were monitored under a 12:12 light-dark (LD) cycle for 14 days (days 8–22). The 12:12 LD cycle was then changed to DD for ten days of additional wheel running monitoring, without interruption (days 23–32). Running wheel cages were 47cm (length) x 27cm (width) x 20cm (height) with a wire mesh floor and a 34cm diameter running wheel. A cotton Nestlet (approximately 2g cotton: Ancare Corporation, Bellmore, NY USA) was provided for nesting material. During the light phase of the 12:12LD cycle the light level at the bottom center of each cage averaged 59±7 lux. During DD a constant dim red monochromatic filtered (Kodak number one) light was provided by three 2-watt fixtures, with light levels measuring below one lux at the top of each cage, to facilitate animal care and observation during DD. Following the 10 day DD period, mice were returned to standard mouse cages with a 12:12 LD cycle and allowed to recover for 3 months before in vivo insulin action was assessed (see below). During this recovery period and throughout the assessment of insulin action, mice consumed a normal chow diet and remained on either 0.1% Reb-A supplemented drinking water or regular drinking water. Mean wheel revolutions per 10-minute interval were measured in the 12:12 LD cycle and in DD, time of peak activity was estimated in 12:12LD as the peak of a best-fit cosine (Acro version 3.5, Roberto Refinetti, Boise State University, Boise, ID USA), and circadian period in DD was estimated using Chi-Square Periodogram (Rhythmwatch software, Minimitter Co, Bend, OR USA).

### Glucose tolerance testing

Mice were housed in standard cages and consumed a normal chow diet for three months before the glucose tolerance test (GTT). Following an overnight fast, mice received an intraperitoneal injection of glucose (1.0 g/Kg body weight) and then tail vein blood was collected (3–5 ul) at 0, 15, 30, 45, 60, and 90 min following the injection. Blood glucose was measured using a hand-held glucometer (Accu-Check, Roche Diabetes Care, Inc). Mice were allowed to recover for 7–10 days before assessing pyruvate tolerance.

### Pyruvate tolerance testing

Following an overnight fast, mice were subjected to a pyruvate tolerance test (PTT). Mice received an intraperitoneal injection of pyruvate (1.0 g/Kg body weight) and then tail vein blood was collected (3–5 ul) at 0, 15, 30, 45, 60, and 90 min following the injection. Blood glucose was measured using a hand-held glucometer (Accu-Check, Roche Diabetes Care, Inc). Mice were allowed to recover for 7–10 days before assessing insulin tolerance.

### Insulin tolerance testing

Following a 6-hour fast, mice were subjected to an insulin tolerance test (ITT). Mice received an intraperitoneal injection of insulin (0.5 U/Kg body weight) and then tail vein blood was collected (3–5 ul) at 0, 15, 30, 45, and 60 min following the injection. Blood glucose was measured using a hand-held glucometer (Accu-Check, Roche Diabetes Care, Inc).

### Caloric intake

Caloric intake was assessed by giving mice a known amount of chow for a given period of time (7 days). Remaining chow in the food bin and bedding was weighed and subtracted from the initial amount of chow and expressed as kcal/day.

### Water intake

Water intake was assessed by giving mice a known volume of water for seven days and then measuring the remaining volume. Our data demonstrate that mice consumed a significantly greater volume of Reb-A treated water compared untreated control drinking water (5.91±0.18 vs. 4.33±0.28 ml/day). Based on the volume of the 0.1% Reb-A solution consumed, mice received approximately 5.9 mg of Reb-A each day.

### Obesity susceptibility

After assessing in vivo insulin action, susceptibility to obesity was assessed by placing all mice on a high fat diet (HFD, 60% kcal from fat) (Catalog # 58126, Test Diets, St. Louis, MO) for two months and assessing body weight and caloric intake. Body weight was assessed prior to and following 2, 4, and 8 weeks of consuming a HFD. Caloric intake was assessed prior to and during the HFD intervention by giving mice a known amount of chow for a given period of time (7 days). Remaining chow in the food bin and bedding was weighed and subtracted from the initial amount of chow and expressed as kcal/day.

### Statistical analysis

Analysis of variance (ANOVA) with repeated measures was used to detect statistically significant effects of Reb-A on body weight and caloric intake and circadian rhythms. ANOVA with repeated measures (time of glucose assessment: 0, 15, 30, 45, 60, and 90 min) was also utilized to detect a significant effect of Reb-A on glucose tolerance, pyruvate tolerance, and insulin tolerance. Following a significant F ratio and inspection of interactions, *a priori* mean comparisons were conducted using Fisher’s least significant difference (LSD) post-hoc test. Data are expressed as means ± SEM and the level of statistical significance was set at p < 0.05.

## Results

### Circadian wheel running activity

**Fig 1:**
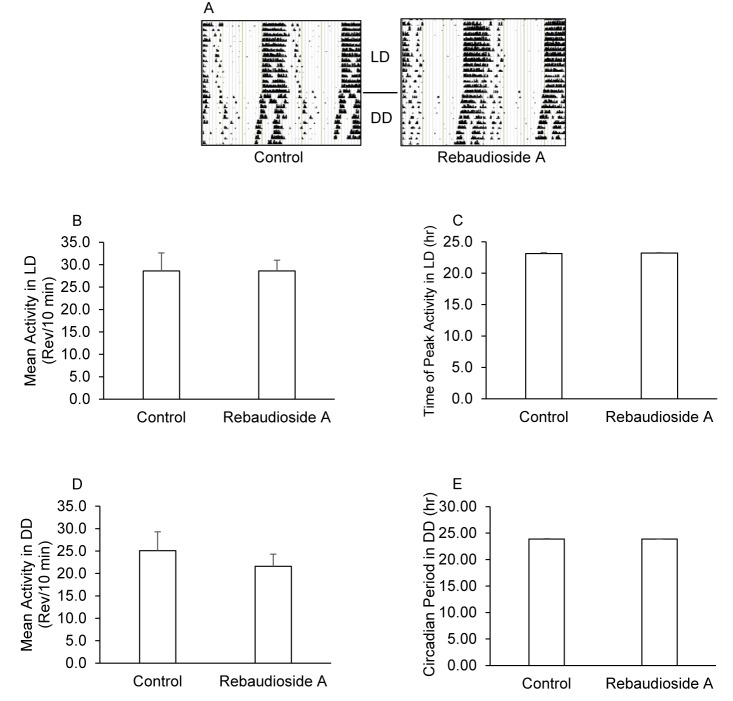
Rebaudioside A treatment does not alter circadian wheel running activity. Effects of Reb-A treatment on circadian running wheel activity rhythms were assayed while mice consumed a standard rodent diet with free-access to running wheels. Panel A shows representative actograms for wheel running activity for 14 days in a 12:12 LD cycle immediately followed by 10 days in constant darkness. The x-axis represents 48 hours of activity, and the Y-axis represents 24 consecutive days from top to bottom. Activity cycles are double-plotted so that each row shows two consecutive days of activity, with the second day in each row repeated as the first day in the next. Panel B shows mean activity levels during the 14 days of LD, and panel C shows the time of peak activity in LD. Panel D shows mean activity levels in DD and panel E shows the circadian period in DD. N = 10 mice per group.

We determined the effect of Reb-A treatment on circadian wheel running activity while mice consumed a normal chow diet ad libitum. As shown in Fig 1, mean activity levels in 12:12 LD (28.6±2.4 vs 28.6±4.0 rev/10 min, P = 0.99) and DD (21.6±2.7 vs 25.1±4.2 rev/10 min, P = 0.49), the time of peak activity in 12:12 LD (23.21±0.08 vs. 23.13±0.14 h, P = 0.64), and circadian period in DD (23.89±0.04 vs. 23.89±0.07 h, P = 0.96) were not statistically different in mice treated with Reb-A compared to control mice. A representative double-plotted actogram for control and Reb-A treated mice is shown in Fig 1A. These data indicate that consuming a 0.1% Reb-A solution does not alter circadian wheel running activity.

### Reb-A treatment does not alter body weight following circadian wheel running activity

**Fig 2:**
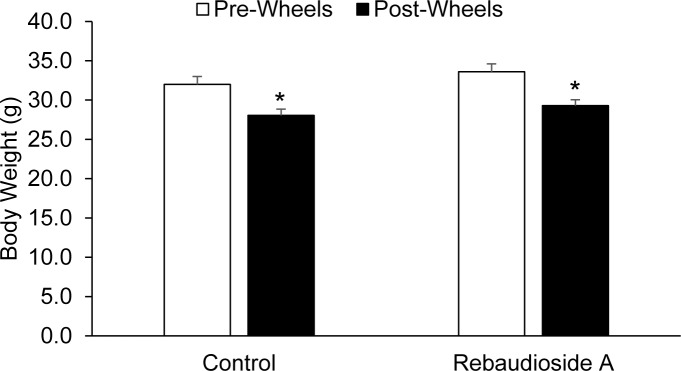
Rebaudioside A treatment does not alter weight loss following circadian wheel running activity. Mice were fed a standard rodent diet for and given free access to a running wheel. Body weight was measured before and after 32 days of wheel running. *, Denotes statistically significant changes in body weight following the wheel running intervention within respective groups of mice. N = 10 mice per group.

We determined the effect of Reb-A treatment on body weight while mice consumed a standard rodent diet (15% of calories from fat) and were given free access to a running wheel for 32 days. As shown in Fig 2, there were no differences in body weight between the mice assigned to the Reb-A group and the mice assigned to control group prior to Reb-A treatment and following wheel running interventions (main treatment effect, P = 0.916). As expected, both the Reb-A and vehicle treated mice lost significant amounts of body weight following 32 days of voluntary wheel running (main time effect, P = 0.0001). However, no differences existed in body weight between the Reb-A and vehicle groups following four weeks of wheel running (LSD post-hoc, P = 0.988) indicating that Reb-A treatment does not hinder the ability of exercise to promote weight loss (Fig 2). In line with similar the body weights, caloric intake was also similar in mice receiving Reb-A treated water compared to mice receiving untreated control water (14.64±0.62 vs. 14.84±0.66 kcal/day).

### Reb-A treatment and insulin action

**Fig 3:**
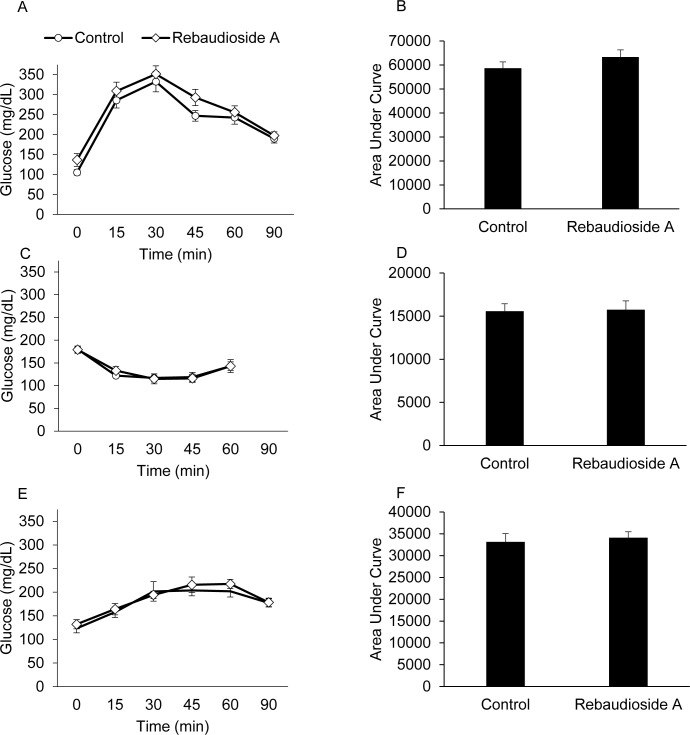
Long-term Reb-A treatment does not alter insulin action. Mice were injected with 1.0 g/Kg glucose (A and B), 0.5 U//Kg insulin (C and D), or 1.0 g/Kg pyruvate (E and F) and blood glucose values were assessed at baseline at 0, 15, 30, 45, 60, and 90 min following the injection. The area under the curve was calculated for glucose levels following the respective injections (Panels B, D, and F). N = 8 mice per group.

*In vivo* insulin action was determined by conducting glucose tolerance, insulin tolerance, and pyruvate tolerance tests. As shown in Fig 3A and 3B, mice that received Reb-A in their drinking water had similar glucose tolerance as vehicle control mice. Next, we conducted insulin tolerance tests to assess peripheral insulin sensitivity. Following insulin administration, blood glucose values were similar in Reb-A treated mice compared to vehicle control mice, indicating no effect on insulin sensitivity (Fig 3C and 3D). Finally, pyruvate tolerance tests were conducted to evaluate the effect of Reb-A on hepatic gluconeogenesis. Fig 3E and 3F reveal no effect of Reb-A treatment on pyruvate tolerance, indicating that hepatic gluconeogenesis is unchanged.

### Reb-A treatment does not increase the susceptibility to diet-induced obesity

**Fig 4:**
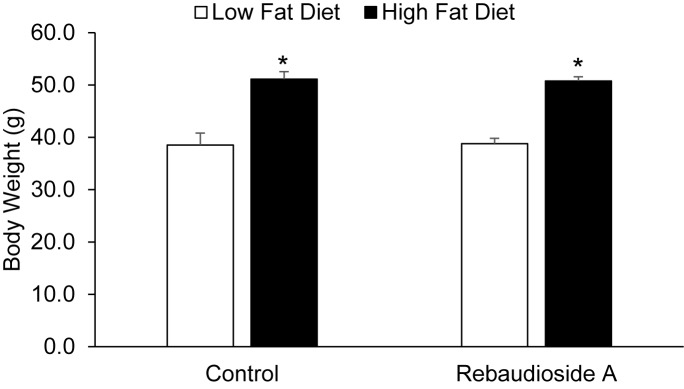
Reb-A does not alter the body weight response to a HFD. Body weight was assessed before and following 18 weeks of a HFD in mice treated with Reb-A or vehicle. *, Denotes statistically significant difference from respective vehicle group by LSD post-hoc analysis (P = 0.0001) conduct after a significant ANOVA main effect for diet (P = 0.0001). N = 8 mice per group.

**Fig 5:**
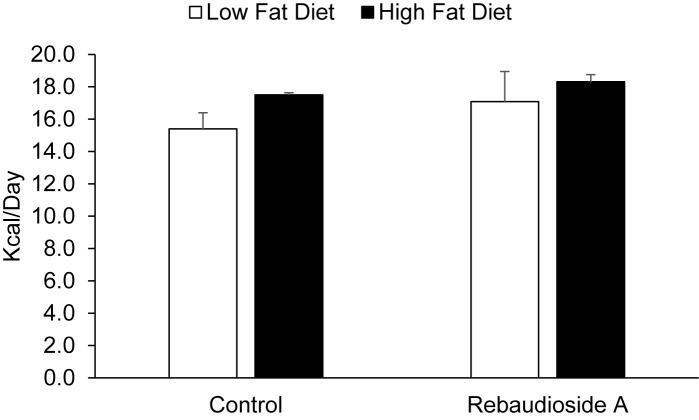
Reb-A does not alter the caloric intake response to a HFD. Caloric intake was assessed before and following 18 weeks of a HFD in mice treated with Reb-A or vehicle. One way ANOVA revealed no significant main effects for Reb-A treatment (P = 0.325) or diet (P = 0.095). N = 8 mice per group.

We determined whether or not Reb-A treatment increases weight gain when consuming a HFD. Body weight and caloric intake were assessed in mice treated with Reb-A or control before and following 18 weeks of a HFD. There were no difference in body weight or caloric intake prior to the HFD intervention, as show in Figs 4 and 5, respectively. As expected, a HFD significantly increased body weight, however, no differences existed between mice treated with Reb-A supplemented water compared to control water (Fig 4).Caloric intake was not different between the Reb-A and control groups either before or following 18 weeks of a HFD, although a trend for increased caloric intake was observed in response to a HFD (P = 0.095).

## Discussion

The consumption of sugar sweetened beverages likely contributes to obesity [8], an increasingly prevalent metabolic disease [1] associated with elevated risk of developing type 2 diabetes. Therefore, consuming artificial sweetened drinks might help reduce an individual’s caloric intake and promote weight loss. Although this seems like a plausible intervention, evidence indicates that consuming artificial sweeteners might actually decrease physical activity [12], promote obesity [9], and, at least for saccharin, cause insulin resistance [10]. Because obesity and insulin resistance are associated with circadian disruption [11], artificial sweeteners may promote weight gain by altering circadian rhythms. Unlike saccharin, the physiological effects of consuming the artificial sweetener Reb-A has not been studied extensively. Therefore, we examined the effects of Reb-A on insulin action, circadian rhythms, and susceptibility to diet-induced obesity.

The present study demonstrates that Reb-A does not promote obesity in mice consuming either a low fat or high fat diet. To date, we are aware of only three published reports that studied the effects of rebaudioside on body weight. Oliveira-Filho et al. [14] demonstrated that 60 days of S. rebaudiana supplementation did not alter body weight in prepubertal rats on a normal chow diet. Likewise, Park and Cha [15] observed less weight gain in mice consuming a high fat diet with stevia supplemented water compared to a high fat diet with sucrose supplemented water. Alternatively, abdominal fat mass appears to be greater in animals fed a stevia supplemented diet compared to a control diet indicating that the artificial sweetener promotes obesity [16]. The present study, similar to Oliveira-Filho et al. [14], clearly shows that prolonged use of Reb-A does not promote obesity. However, it should be noted that the present study did not account for the cognitive impact non-caloric sweeteners have on subsequent caloric intake. Classic Pavlovian experiments by Davidson et al. show that pre-exposure to sweet taste with saccharin disrupts satiety signals and leads to greater caloric intake when saccharin is replaced with glucose [17]. Nonetheless, the present study argues against the idea that Reb-A promotes obesity when the ability to associate sweet taste and caloric content is not disrupted and animals are given free access to chow and water that is identical in caloric content. Despite mice receiving approximately 6 mg of Reb-A per day we can’t be absolutely certain that active rebaudioside metabolites were consumed because we did not assess their levels in the Reb-A treated water [18]. Our study is further limited by only studying one dose of Reb-A, as higher Reb-A concentrations may have impacted adiposity and insulin sensitivity.

The observation that acute ingestion of sucralose prior to an oral glucose tolerance test promotes insulin resistance suggests that chronic consumption of artificial sweeteners is ill-advised for individuals at risk for type 2 diabetes [19]. The present finding that chronic consumption of Reb-A does not impair insulin action is different from other studies in animals and humans demonstrating that artificial sweeteners are associated with metabolic disease [10,20–22]. Although the acute ingestion of either aspartame or saccharin does not seem to alter glucose homeostasis [23], Suez et al. [10] demonstrated that chronic consumption of saccharin resulted in glucose intolerance, a process associated with changes in the gut microbiota. In contrast to Suez et al., the present study showed no deleterious effect of long-term Reb-A consumption on glucose tolerance, insulin tolerance, or pyruvate tolerance. Our findings indicate that stevia based artificial sweeteners do not impact glucose metabolism, an observation that appears to be distinct from other commercially available artificial sweeteners.

The present study is the first to examine the effect of consuming Reb-A on circadian rhythms. In fact, the role of commonly used artificial sweeteners on circadian rhythms is not well established as we are aware of only one report that indicates saccharin disrupts circadian sleep-wake cycle and decreases activity in mice [12]. Here, we clearly demonstrate that Reb-A does not alter circadian rhythmicity. The importance of this finding is underscored because numerous studies showing that disruptions of circadian rhythms increase the susceptibility to obesity [24,25]. Since shift workers have greater rates of obesity and routinely experience circadian disruption [26,27], using an artificial sweetener that causes further rhythm disruption might impede weight loss and actually promote obesity in this population.

## Conclusions

The present study demonstrates that long term Reb-A treatment does not promote obesity or alter insulin action. Further, we show that Reb-A treatment does not disrupt circadian rhythms. These finding indicate that Reb-A may provide a method to reduce energy intake without reducing food intake, thereby creating an energy deficit that would facilitate weight loss.

## Supporting information

S1 File Reynolds et al data. (ZIP)
